# Solid‐state dosimeters have rendered ionization chambers obsolete for routine diagnostic X‐ray quality control

**DOI:** 10.1002/acm2.70657

**Published:** 2026-06-10

**Authors:** Allen R. Goode, Kevin A. Wunderle, David W. Jordan

**Affiliations:** ^1^ Department of Radiology and Medical Imaging UVA Health Charlottesville Virginia USA; ^2^ Department of Radiology The Ohio State University Columbus Ohio USA; ^3^ Department of Radiology University Hospitals Cleveland Medical Center, Case Western Reserve University School of Medicine Cleveland Ohio USA; ^4^ Department of Radiology VA Northeast Ohio Healthcare System, Case Western Reserve University School of Medicine Cleveland Ohio USA

**Keywords:** diagnostic, dosimetry, measurement, X‐ray

## Abstract

Today's diagnostic medical physicist finds a broad variety of instruments available to make the numerous quality control (QC) measurements needed for routine equipment evaluations. One of the distinguishing features of different options is the availability of multi‐purpose solid‐state detectors (SSD) and application‐specific ion chambers (IC) that can be used for measurements of the various modality equipment types. Since these measurements are the “bread and butter” of diagnostic physics work and the foundation of quality and safety for patients, the choice will have profound implications for many aspects of the physicist's work. In this debate, we explore the roles of SSD and IC instruments through the insights and perspectives of two highly experienced and accomplished diagnostic medical physicists.

## 1 INTRODUCTION

Today's diagnostic medical physicist finds a broad variety of instruments available to make the numerous quality control (QC) measurements needed for routine equipment evaluations. One of the distinguishing features of different options is the availability of multi‐purpose solid‐state detectors (SSD) and application‐specific ion chambers (IC) that can be used for measurements of the various modality equipment types. Since these measurements are the “bread and butter” of diagnostic physics work and the foundation of quality and safety for patients, the choice will have profound implications for many aspects of the physicist's work. In this article, we explore the roles of SSD and IC instruments through the insights and perspectives of two highly experienced and accomplished diagnostic medical physicists as they debate whether “solid‐state dosimeters have rendered ionization chambers obsolete for routine diagnostic X‐ray quality control.”

Writing for the proposition, Allen Goode, M.S. is Chief Diagnostic Medical Physicist at the University of Virginia. He received his Master's degree in Biomedical Engineering from the University of Virginia and is certified in Diagnostic Medical Physics by the American Board of Radiology. He serves as Cochair of the University of Virginia Clinical Radiation Safety Committee. He is active in numerous AAPM committees and has co‐authored three AAPM reports. His research interests include radiation dosimetry, instrumentation, and patient and staff radiation protection. He also leads the development and delivery of the physics curriculum for radiology residents at the University of Virginia.

Writing against the proposition, Kevin Wunderle, Ph.D. is Professor of Radiology at Ohio State University. He received his Ph.D. in Medical Physics from Wayne State University and is certified in Diagnostic Medical Physics by the American Board of Radiology. He has extensive clinical and research experience in interventional fluoroscopy, and his research interests include radiation dosimetry and patient and staff radiation protection. He is the Vice Chair of the AAPM Imaging Physics Committee and has co‐authored three AAPM reports. He is a Fellow of AAPM and ACR and is active in the National Council on Radiation Protection and Measurements and the International Electrotechnical Commission. He also serves on the curriculum committee for the Ohio State diagnostic medical physics residency program.

## 2 OPENING STATEMENTS

For the proposition: Allen Goode, MS

Modern diagnostic X‐ray QC requires rapid and accurate assessment of multiple beam parameters, often within tight clinical time constraints. While ionization chambers (ICs) have historically served as the foundation of basic dosimetry measurements, solid‐state dosimeters (SSDs) now provide a more comprehensive and efficient solution for routine QC measurements. SSDs offer a complete solution, often with additional beam parameters compared to ICs, without the need for biasing. Multipurpose SSDs have become ubiquitous in the clinic for collecting medical physicists’ X‐ray beam parameters associated with radiographic or fluoroscopic exposures. While many vendors supplying tools to diagnostic medical physicists offer both SSDs and ICs, I consider an SSD to be the “Swiss Army Knife” style device and a more complete option when selecting a sensor to test diagnostic X‐ray systems. These sensors, while evaluating air kerma as their IC counterparts do, also offer a plethora of technical parameters from a single shot or fluoroscopic event. From a single exposure, many SSDs will capture multiple variations of kilovoltage (kV), including peak, and average, as well as pulse time, pulse rate, number of pulses, half value layer (HVL), total filtration, as well as air kerma and air kerma rate. Furthermore, many of these parameters can be visualized graphically by the electrometer subsystems (or computer), making for a powerful and complete diagnostic tool. On most X‐ray unit evaluations, more information is needed than simple air kerma or rate, why not carry a tool that can do it all, and reduce setup and switching time to and from an IC?

SSDs are also simply more robust than ICs are. When using an SSD, I am not worried about damage to a fragile outer shell of the sensor. Most SSDs are encased in a metal housing, which protects the inner diodes from damage during hasty inspection tasks and the “accidental drop to the floor.” Speaking of accidents, for those physicists that travel in regions with extreme temperatures, and leave their equipment in the car, there is a necessary “equilibration period” for the IC. While some IC electrometers can correct for temperature and pressure, there is no doubt that harsh temperature conditions are a concern for IC's whereas SSDs are far less sensitive to temperature effects. In fact, many electrometer units can apply any environmental corrections needed. While the energy dependence of SSD's is strong, their ability to self‐correct based on energy and environmental conditions makes for a flat response across the diagnostic energy spectrum and testing conditions.

In mammography testing, many systems with SSD's include the ability to compensate for anode/filter combination in mammography, making SSDs invaluable for newer complex anode/filter combinations in the lower kV settings. In fact, the auto HVL features provided by SSDs have significantly cut our inspection time for mammography machines.

SSDs often have the unique ability to reject backscatter during measurements. This scatter attenuating capability, typically accomplished with a high Z backing material, makes SSD measurements near equivalent to in‐air measurements of their IC competitors. For fluoroscopy air kerma rate measurements, this scatter rejection makes SSDs superior to ICs for many measurements needed for the regulatory checks during equipment performance evaluations. Furthermore, as we move to lower air kerma rates of fluoroscopy, measuring those rates with a chamber becomes problematic due to signal to noise limitations in the IC bias cable, leakage current or possibly electrometer drift around zero baseline. Often, physicists are concerned about high Z material makeup of SSDs being used in the field of view of fluoroscopy for air kerma measurements. While this would seemingly be a valid concern, as long as the SSD is out of the dominant or measuring field controlling the Automatic Dose Rate and Image Quality (ADRIQ) controls, or left in the same position for all measurements‐ the effects of the high‐Z backing in the SSD are the same for all measurements. In fact, localized processing on many of the newer advanced fluoroscopes is detecting objects in the field of view. Having a field of view with only plastic or copper sheets and a chamber that has almost no attenuation, is dissimilar to what the fluoroscopes image clinically, and may cause unwanted results.

Lastly, most vendor service engineers use the smallest most compact system they can carry, which typically is that of a dosimetry kit containing an SSD. In situations where you are trying to compare air kerma, or investigating errors with a kerma area product meter, it makes more sense to use a similar device as the service engineer for a cross‐validation – instead of scatter correcting data from an IC which could introduce troubleshooting inconsistency.

Modern clinics require nearly 100% uptime; speed is essential to the clinic. Clinical medical physicists need to get in and out of an inspection and not continually switch sensors. If you have to head to a diagnostic inspection and can only pick one device, I would choose an SSD. SSDs align better with modern clinical workflows, are fast, reliable, and are less prone to noise or environmental conditions. I would choose the “Swiss Army Knife” over the limited utility “steak knife” or IC. While ICs may remain the gold standard for absolute dosimetry, seldom is this primary standard needed for routine QC.

Against the proposition: Kevin Wunderle, PhD

Let me first acknowledge the obvious: SSDs, which have been present in clinical use for quite some time, are undeniably convenient. It is a seductive proposition for a medical physicist to place a single, compact device on a patient table, make one exposure, and instantly receive a readout for kVp, air kerma, half‐value layer (HVL), and equivalent filtration. I do not deny their utility or efficiency. However, in the rigorous practice of diagnostic medical physics, convenience must never supersede accuracy and certainty.

The fundamental flaw in this proposition lies in the assumption that all SSDs are universally reliable, interchangeable, absolute dosimeters. They are not. There are documented differences in the performance of SSDs across different vendors, and often variations even within a single vendor's product line depending on the generation of the equipment. When relying solely on these devices, a physicist is not dealing with a unified, universally standardized physical reaction, but rather with proprietary “black boxes”.

Unlike a direct measurement of charge in a volume of air, the accuracy of an SSD multi‐sensor relies heavily on complex calibration curves and internal, manufacturer‐specific algorithms. These devices calculate dose and beam quality by comparing the incident radiation to a presumed, idealized beam spectrum programmed into their firmware. When clinical reality matches laboratory calibration, the results are generally excellent. But physics in the clinic is often not ideal.

Because these algorithms are empirically derived and rely on discrete data points, they are inherently vulnerable to discrete algorithmic errors. A flaw within a specific calculation pathway may yield non‐uniform errors across its operational range. This introduces a silent, unpredictable variable nearly impossible to anticipate during routine use. Indeed, this vulnerability exists for all look‐up table‐based dose estimates that abstract away from a directly measurable physical quantity, like charge collected in a volume of air. This behavior stands in stark contrast to an ionization chamber, governed by physical interactions. Provided the chamber is functionally intact and properly calibrated, it yields results that can be implicitly trusted and understood from first principles.

Recent studies evaluating modern SSDs against reference ionization chambers demonstrate reasonably good accuracies for standard diagnostic measurements.^1^, ^2^, ^3^ For example, investigations observe average air kerma underestimations of only 3% to 4% at common potentials (e.g., 70 kV). While respectable, this baseline accuracy is achieved under straightforward conditions, such as 2.8 mm Al total filtration, mimicking a standard RQR5 calibration beam. Outside these factory‐calibrated ideals, accuracy predictably degrades. Because an SSD's response is dictated by the assumed beam spectrum, its algorithms can be misguided by unique target‐filter combinations yielding spectra outside calibration conditions. Manufacturers may claim a broad ±5% acceptable uncertainty, but this strictly assumes adherence to their provided beam quality ranges.

This vulnerability is particularly evident when evaluating modern fluoroscopes that utilize dynamic spectral filtration. These systems frequently insert varying thicknesses of copper and aluminum, or alternative materials such as gold or titanium. When these filtration conditions deviate from the SSD's explicit calibration, relative errors increase, creating substantial measurement uncertainty. Furthermore, SSD accuracy may depend on precise physical positioning. For some SSDs, if they are improperly aligned with respect to the anode heel effect, they may yield erroneous results. They may also be sensitive to scatter; an unexpected scatter profile can drastically alter the signal in the energy‐dependent diodes, yielding highly precise, but fundamentally inaccurate, data.

Contrast this uncertainty with the venerable ionization chamber. Assuming a given chamber is in good working order, calibrated, and appropriate for the measurement being made, its operation is fundamentally straightforward and extraordinarily stable. An ionization chamber provides a direct physical measurement of ionization in air, completely independent of hidden algorithms or assumed spectra. Crucially, ionization chambers are also effectively free of beam quality dependence across the diagnostic energy range. They will accurately measure air kerma regardless of whether the beam is heavily or lightly filtered. Furthermore, they generally exhibit virtually no angular dependence, making them immune to the precise positioning errors and off‐axis variations that often afflict flat SSD arrays.

Consider the following clinical scenario: a medical physicist encounters a failing result, such as an out‐of‐tolerance HVL measurement, during routine QC using an SSD. A field service engineer follows up, performs their own testing with a different SSD, and declares the system normal. How is this standoff resolved? How does the physicist determine if their measurement indicates a true hardware or software issue requiring corrective action, or if their SSD's algorithmic assumptions were simply violated? You cannot troubleshoot a black‐box algorithm with another black‐box algorithm. To answer this question with confidence, you must return to first principles and ionization chamber measurements.

SSDs are fantastic tools for rapid data collection. But to claim they are “all that are needed” for routine QC strips the medical physicist of their most foundational, reliable, and truth‐telling instrument. Because of their energy dependence, susceptibility to radiation damage, angular sensitivity, and reliance on assumed spectra, SSDs simply cannot stand alone. Therefore, I respectfully encourage the readers of JACMP to reject this proposition.

## 3 REBUTTAL

For the proposition: Allen Goode, MS

Dr. Wunderle's argument for ICs is not without merit, and he makes several great points. However, in his argument for continued reliance on ICs he indicated several caveats and shortcomings of SSDs which need addressing. First, in his statement he indicates that the “flaw in this proposition lies in the assumption that all SSDs are universally reliable, interchangeable, absolute dosimeters.” I will point out that a great number of field service engineers from the majority of vendors I have worked with use SSDs to setup their systems in the field. Why? For their simplicity, reliability and accuracy for clinically relevant tolerances.

In his argument for continued reliance on ICs is also the notion of the “black box” with respect to firmware, software and the hidden features of SSDs that may not be known or adjustable. Dr. Wunderle's first reference, Maruyama et al, aptly points out, under scrutiny of reference sensors, relative differences in the measurement air kerma of only a few % for all but the most heavily filtered beams were shown. Routine diagnostic QC is not an exercise in primary standards dosimetry, but in detecting clinically meaningful deviations in system performance. Modern SSDs achieve agreement within a few percent of reference measurements, a level of accuracy that exceeds what is necessary for effective QC while offering substantial gains in efficiency and usability.

With respect to energy dependence and performance outside calibration conditions, Dr. Wunderle asserts that SSDs face significant challenges when it comes to highly filtered beams such as those on modern fluoroscopes. I disagree and think that this is where SSDs maintain acceptable agreement; the ability to evaluate the spectrum and therefore beam quality to correct measurements on the fly, clinically. Lin et al evaluated SSDs with an IC at common beam qualities in interventional imaging, and the results indicate solid agreement with ICs for HVLs (calculated vs. automated) at RQR5 beam conditions.^4^ Even though this work showed that for higher kV's and more heavily filtered beams the accuracy of automated HVLs from SSDs decreases slightly in the higher keV extended areas, the data showed SSDs are capable of estimating the spectral properties of heavily filtered beams‐ such that they can be used for inherent corrections.

While Dr. Wunderle asserts that ICs provide ground truth dosimetry due to their simplicity, it's important to acknowledge, ICs still require a priori beam‐quality dependent corrections, similar to SSDs. As AAPM TG‐61 points out, energy and beam corrections from electrometer and IC need to occur together or be accounted for separately.^5^ Furthermore, even among ICs, energy response is not universal. Chambers from different manufacturers exhibit small but measurable differences in response due to variations in wall composition, geometry, and construction. Even with proper temperature and pressure corrections, chamber‐specific calibration and beam quality corrections are required, undermining the notion of a single, universally consistent “ground truth” measurement. The so‐called “energy‐independent” ICs exhibit measurable variation in response across the diagnostic energy range, typically on the order of a few percent depending on beam quality. Manufacturer specifications for a commonly used chamber, for example, report variations up to ±4% across relevant photon energies, underscoring the need for beam‐quality‐dependent corrections.

Lastly, Dr. Wunderle presents a “standoff” situation, where 2 SSDs disagree over an HVL measurement. This is a rare, albeit difficult situation, which must be resolved correctly. In most instances, a 3rd device is used for comparison, to “break the tie”, and Dr. Wunderle indicates it in fact must be an IC with less unknowns. I disagree, and it is likely an additional SSD will be absolutely adequate. Even in this situation, an IC is not necessary; if the measurement is that close to failing, neither of the sensors are the issue to focus on.

Given the complex spectral filters Dr. Wunderle discusses, which are used to decrease the skin dose on our patients, air kerma rates from fluoroscopes are becoming lower and lower. At low dose rates, IC signals are more susceptible to noise and leakage effects whereas SSDs still produce a robust measurable, digital signal. SSDs offer superior performance at low air kerma rates, clinical efficiency, and the benefit of multi‐parameter capability. For this reason, SSDs have supplanted ICs as the go‐to sensor in the field.

Against the proposition: Kevin Wunderle, Ph.D.

My esteemed colleague makes a compelling case for efficiency, characterizing the solid‐state detector (SSD) as the “Swiss Army Knife” of diagnostic medical physics. While I appreciate this analogy, it inadvertently highlights the inherent trade‐offs of such devices. A Swiss Army Knife is a tool of ultimate convenience, excellent for a wide variety of general tasks, but it is not a precision instrument. For clinical dosimetry, patient safety, protocol optimization, and regulatory compliance, we often require the exactness of a dedicated tool. The ionization chamber (IC) is not merely a “limited utility steak knife”; it is a reference‐grade instrument governed by physical laws rather than proprietary approximations.

The opposing argument heavily praises the “auto‐correction” and “backscatter rejection” capabilities of SSDs. However, these features are precisely what introduce the hidden uncertainties I described in my opening statement. The claim that SSDs achieve a “flat response” via auto‐correction assumes the device correctly identifies the beam spectrum in the first place, one for which it is also calibrated. When evaluating modern interventional systems with dynamic spectral filtration, these algorithmic assumptions can fall short.

Furthermore, the discussion regarding the high‐Z backing of an SSD for fluoroscopy QC merits careful consideration. To avoid driving the Automatic Dose Rate and Image Quality (ADRIQ) logic with a highly attenuating detector, my colleague suggests placing the SSD “out of the dominant or measuring field.” However, moving the detector off the central axis introduces complex dosimetric variables. By measuring off axis, the device becomes potentially subjected to the anode heel effect, which can significantly alter both the beam intensity and the spectral quality compared to the central ray. Additionally, the scatter distribution in these peripheral locations can vary unpredictably. A radiolucent IC can be placed centrally within the beam without artificially driving the ADRIQ system, eliminating the need to compromise on measurement positioning.

The consequences of relying solely on algorithmic approximations are not merely theoretical. In my own clinical practice, I have evaluated several radiographic/fluoroscopic units where one of the most popular brands of SSDs yielded failing half‐value layer (HVL) results during “convenient” direct measurements, including during acceptance testing. This initially suggested a regulatory compliance failure. However, when the HVL was carefully reassessed using an ionization chamber, the systems passed. Relying exclusively on the SSD in that scenario would have resulted in unnecessary downtime and unwarranted service interventions.

This real‐world example directly challenges my colleague's suggestion that physicists should use an SSD simply to match the vendor service engineer's equipment, thereby avoiding “troubleshooting inconsistency.” If both the engineer and the physicist use the same “black‐box” SSD, they share the identical algorithmic blind spots. In the HVL scenario described above, using identical equipment would have merely resulted in us consistently agreeing on the wrong number, exacerbating a non‐issue. While standardizing tools can be helpful, the core mandate of the medical physicist is independent, rigorous verification. Consistency between two identical devices does not guarantee dosimetric accuracy. When troubleshooting complex issues, an independent measurement methodology, such as an IC, is invaluable for uncovering the true nature of the problem.

Finally, while an IC may require a brief period to equilibrate to ambient temperature and pressure when moving between dissimilar environments, this is a minor logistical hurdle. Planning a short equilibration period while setting up a phantom and other equipment is a minuscule price to pay for dosimetric accuracy.

We all operate under tight clinical time constraints, and utilizing an SSD for rapid data collection is an entirely reasonable workflow. However, to declare ionization chambers “obsolete” is to accept the inevitability of occasional, unidentifiable errors without a gold standard for verification. Accuracy must remain our primary objective. I respectfully urge the readers of JACMP to recognize the indispensable value of the ionization chamber and reject the proposition.

## AUTHOR CONTRIBUTIONS

All authors drafted, revised, and gave final approval for the manuscript.

## CONFLICT OF INTEREST STATEMENT

The authors declare no conflicts of interest.
